# The Prevention of Acute Rheumatism
*From a paper delivered to the Bristol Medico-Chirurgical Society on 9th January, 1957.


**Published:** 1957-07

**Authors:** C. Bruce Perry

**Affiliations:** Professor of Medicine, University of Bristol


### THE PREVENTION OF ACUTE RHEUMATISM*

BY

C. BRUCE PERRY, M.D., F.R.C.P.

*Professor of Medicine, University of Bristol*

If the title of this paper suggests that the mystery of acute rheumatism is solved, it would be far from true, for the factors which condition the occurrence of this disease still elude us.

### SOCIAL INCIDENCE

It has long been known, however, that acute rheumatism is a disease which shows a striking social incidence. The researches instituted in Bristol by Dr. Carey Coombs confirmed, in 1930, that it was predominantly an urban as opposed to a rural disease, the incidence in Bristol being about four times that in the surrounding counties[1]. In Bristol itself there was an even greater variation in different parts of the city which we were later able to show was clearly related to both overcrowding and poverty[2, 3, 4]. Which of these is the more important we do not know. Overcrowding might be the crucial factor by facilitating infection, or poverty might result in a deficiency of some essential food factor. It may be that the recent change in social conditions is responsible for the rapid decline in incidence and severity of acute rheumatism which has taken place during the last thirty years. In 1925, according to the Registrar General's returns, rheumatic fever was responsible for 1885 deaths in England and Wales, whereas in 1955 the comparable figure was 227. The same trend is revealed by figures obtained from notifications of acute rheumatism. Since October 1947 the disease has been notifiable in Bristol and certain other parts of the country. In 1948, the first full year of notification, there were 89 confirmed cases of acute rheumatism notified in Bristol for every 100,000 school-children; in 1956 the incidence had fallen to 27.5 per 100,000.

### STREPTOCOCCAL INFECTION

We should, however, perhaps note that this decline is paralleled by a decline in the severity of streptococcal illnesses, for instance deaths due to scarlet fever. For it is now as certain as anything can be in medicine, that the Lancefield Group A beta-haemolytic streptococcus is the precipitating cause of rheumatic fever. How the streptococcus causes rheumatic fever is still obscure and why only ½–5 per cent. of persons afflicted with a haemolytic streptococcal infection subsequently develop rheumatic fever, and why children aged 5 to 15 do so more frequently than adults or younger children remains one of the major mysteries of medicine. But we can now say "No haemolytic streptococcal infection, no acute rheumatism".

The evidence for this is four-fold. First, from clinical observation there is the now well-known phenomenon of a streptococcal sore throat followed after a "silent" interval by rheumatic fever; this has been particularly clearly studied in the long-term hospital schools for rheumatic children. Secondly, streptococcal antibodies have been recognized in the blood of patients with rheumatic fever. Todd first described how a high titre of anti-streptolysin O occurs as frequently in patients with acute rheumatism as it does in patients convalescent from scarlet fever; there is a similar rise in the titre of the other antibodies—anti-streptokinase and anti-hyaluronidase. Thirdly, there is the evidence obtained from epidemics of rheumatic fever; these have been observed in schools, and training depots where young people are crowded together, and they are

* From a paper delivered to the Bristol Medico-Chirurgical Society on 9th January, 1957.

always preceded by an epidemic of haemolytic streptococcal infections. Finally, we have learnt that if we prevent haemolytic streptococcal infections we abolish recurrences of acute rheumatism.

### PREVENTION OF RECURRENCES

It is well recognized that acute rheumatism is a disease in which recurrences are very common and that the rheumatic patient's greatest risk of such a recurrence is during the first few years following the initial attack. This gives us our first clue as to how we should tackle the problem of prevention. If we could prevent streptococcal infections in rheumatic children for five years after the initial attack or during school life we should prevent the majority of recurrences, and it is of course the recurrences that are of major importance in producing serious and crippling rheumatic heart disease. There is now ample proof that this is possible in either one of three ways.

### 1. *Prophylactic sulphonamide*

We can keep all children who have had acute rheumatism on continuous prophylactic sulphonamide, and this has been done on the largest scale for the longest time. Published results up to date have recently been summarized[5]. In nearly fifteen hundred "patient years" the incidence of rheumatic recurrences was reduced to nearly a tenth of that in carefully matched untreated controls. But sulphonamide prophylaxis is not without its hazards. It may, fortunately rarely, produce skin rashes and blood dyscrasias; these usually occur in the first few weeks and most people therefore recommend that prophylactic administration of sulphonamides should be started while the patient is in hospital with the acute attack of rheumatic fever so that he can be under close observation during the most critical time. In a small experiment we did some years ago we found that we were afraid to continue to give the drug in a certain proportion of cases because of skin rashes and changes in the blood count—although we have since been told that we were too timid. Also we found that treated patients did not gain as much weight or seem quite so well as controls. Finally there is some—perhaps slight—evidence for the risk of resistant streptococci emerging. But the method is simple and cheap, suitable dosage being 1·0 g. of sulphadiazine daily or 0·5 g. for a child under seven. It needs, of course, full co-operation of the patient so that he never misses a dose, because one day without the drug is quite sufficient for streptococcal infection to occur with a subsequent relapse of acute rheumatism.

### 2. *Oral penicillin*

For this reason attempts have been made to use other measures and here penicillin has proved to be unequalled. Penicillin may be used by mouth in the shape of tablets of Penicillin G, 200,000 units twice daily, or perhaps more efficiently of phenoxymethyl penicillin (Penicillin V) 120 mg. twice daily. Of course penicillin prophylaxis of rheumatic recurrences has a much shorter history and has been used less extensively than sulphonamides, but the results are impressive[6, 7, 8]. The collected figures for seven reported series shows an incidence of 8·7 per cent. recurrences per patient year in controls and only 0·6 per cent. in treated children. There is the same problem about co-operation of the patient, but toxic effects are extremely rare and penicillin-resistant haemolytic streptococci have never appeared. There is a risk of producing penicillin-resistant straphylococci but these organisms are now so widespread that the danger is perhaps not serious.

### 3. *Penicillin injections*

A different approach is to administer long-acting repository penicillin—benzathene penicillin—by monthly intramuscular injection. An injection of benzathene penicillin (Penidural L-A) 1·5 mega units (5 ml.) once a month produces blood levels which are adequate for prophylaxis. It has the disadvantage of all injections in that they may produce local pain and general malaise and fever, but with the new preparation introduced last autumn the incidence of these unpleasant side effects has greatly diminished. Moreover, results are perhaps better than with oral penicillin[9]. Incidentally, while sulphonamides are of little value in eradicating the haemolytic streptococcus from carriers, penicillin by mouth or injection is extremely efficient, provided it is continued for long enough; a monthly injection of benzathene penicillin is perhaps the most effective of all[10].

### *Duration of Prophylaxis*

We can thus prevent rheumatic patients from developing recurrences.—but for how long should prophylaxis be continued? Some enthusiasts in the American Heart Association say for life. This may prove in the end to be necessary but it is a severe sentence. Perhaps a wiser plan at the moment is to advise some form of continuous prophylaxis for five years after the last attack of acute rheumatism or until the age of 15 years, whichever is the longer. If so we should always remember to re-institute prophylaxis whenever the patient is exposed to excessive risk of infection with the haemolytic streptococcus. These occasions are:

(1) When any individual in the patient's household develops a haemolytic streptococcal infection.

(2) When the patient is admitted to any hospital ward.

(3) When he enters any semi-closed community such as a camp or institution where haemolytic streptococcal infection is likely to be prevalant.

Such intermittent prophylaxis for special exposure might well be maintained for life.

### PREVENTION OF FIRST ATTACK

So much then for the prevention of recurrences. Can we do anything to prevent a patient who has a haemolytic streptococcal sore throat from subsequently developing acute rheumatism? Our evidence that we can is largely derived from work carried out on the U.S. armed forces, but it is fairly conclusive. Early results were encouraging and indicated that any treatment which rapidly and completely eliminates the haemolytic streptococcus from the throat of the patient, provided that it is instituted sufficiently early in the course of the disease, will prevent subsequent acute rheumatism[11, 12]. How can we do this? It requires more than merely "treating" the sore throat or even suppressing the organisms. Sulphonamides for this are useless both in preventing rheumatism and in eliminating the streptococcus. Penicillin properly given is very effective; inadequate dosage is, however, as useless as sulphonamides. A single dose, however large, of a short-acting preparation suppresses the streptococcus for a time only. What is needed is adequate blood levels for ten days. How we are to achieve this depends of course on the type of penicillin we use. Oral penicillin is effective for a few hours, procaine penicillin for a day or so, procaine penicillin in oil for some days, and benzathene penicillin for weeks.

### *Penicillin dosage*

We can thus gain our end equally well (*a*) with large doses of oral penicillin (Penicillin G, 200,000 units four times a day, or Phenoxymethyl penicillin—Pencillin V—120 mg. four times a day) for ten days, but not if we use an inadequate dose or give it for too short a time; (*b*), by several injections of procaine penicillin in oil (procaine penicillin in aluminium monostearate 500,000 units every other day for four doses); or (*c*), by one large injection of benzathene penicillin, 1·5 mega units (*Penidural L.A.* 5 ml.)—or perhaps preferably a combination of benzathene with crystalline and procaine to give high initial levels, for instance 300,000 units of Penicillin G, 300,000 units of procaine penicillin and 600,000 units of benzathene penicillin (*Penidural all-purpose*). And we can use the same measures to prevent a recurrence after a sore throat in a rheumatic child who has not received prophylaxis or where prophylaxis has for some reason or other failed. In rheumatic children where there is a 50 per cent. risk of a streptococcal sore throat giving rise to a relapse of acute rheumatism the results of this prophylaxis are very encouraging[13]. In one of the earlier studies half the controls had a relapse and only 6 per cent. (two) of the treated cases, and both of these had a relapse of the streptococcal infection, i.e. the organism was probably not completely eradicated. In a series of patients with sore throats some of whom received no treatment, some sulphonamide and some penicillin, even when treatment was delayed until the ninth day of the illness, only 3 of 420 patients treated with penicillin developed rheumatic fever, i.e. 0·7 per cent., whereas 3·6 per cent. of 220 controls and 5.2 per cent. of 230 treated with sulphonamides developed rheumatic fever subsequently. Is there any alternative to penicillin if perchance a patient is sensitive to penicillin? Nothing so effective although the tetracyclines have some use as shown by Catanzaro and his colleagues[14].

*Recognition of streptococcal sore throat*

If we are then, to treat efficiently all cases of haemolytic streptococcal pharyngitis in order to prevent first attacks of acute rheumatism, how are we to recognize them? Clearly we do not want to employ such energetic therapy for all and every sore throat of which perhaps 80 per cent. will not be due to the haemolytic streptococcus. A throat swab is of course a help, but the presence of haemolytic streptococci in the swab does not prove that they are the causal organisms as up to 25 per cent. of normal children are carriers. On the other hand, a negative throat swab, provided it is taken properly and cultured without undue delay, almost certainly excludes haemolytic streptococcal infection. For these reasons the clinical features become important, and some people think that it is possible to diagnose a streptococcal sore throat accurately in a fair proportion of cases on clinical grounds. The important features are:

(1) The rash of scarlet fever.

(2) A red oedematous throat with exudate and enlarged cervical glands.

(3) The occurrence of abdominal pain, headache and vomiting.

(4) If a white blood count is possible, a polymorphonuclear leucocytosis.

(5) The existence of haemolytic streptococcal infection or its complications—e.g. otitis media—in the patient's household.

Features which make a haemolytic streptococcus unlikely as the cause are the occurrence of coryza, cough or hoarseness. If in doubt perhaps the wisest course is to take a throat swab and have it plated as soon as possible and to start treatment with penicillin but to continue only if the swab proves positive.

### SUMMARY

To sum up then, to prevent relapses of rheumatic fever we can employ continuous prophylaxis with sulphonamides, or with oral or injected penicillin. This must be continuous and should be continued for five years after the last attack or until the patient is 15, whichever is the longer. This treatment should be re-instated later in life whenever the patient is exposed to special risk. We can hope to prevent first attacks of rheumatic fever and relapses where prophylaxis has failed by adequate treatment of all haemolytic streptococcal infections so that the organism is eradicated from the throat. This we can achieve by oral or injectable penicillin in doses that will give an adequate blood level for at least ten days, or if this is contra-indicated with a tetracycline, also for ten days.

### REFERENCES

1 Savage, W. G. (1931). *B.M.J. Supp.*, **2**, 37.

2 Perry, C. B. and Roberts, J. A. F. (1937). *B.M.J. Supp.*, **2**, 154.

3 Daniel, G. H. (1943). *Brist. Med-Chi. J.*, **LX**, 13.

[4] Perry, C. B. (1944). *Brist. Med-Chi. J.*, **LXI.**
[5] Stollerman, G. H. (1954). *Am. J. Med.*, **17**, 757.
[6] Mortimer, E. A. and Rammelkamp, C. H. (1956). *Circulation*, **14**, 1144.
[7] Kohn, K. H., Milzer, A. and MacLean, H. (1953). *J.A.M.A.*, **151**, 347.
[8] Gale, A. H., Gillespie, W. A. and Perry, C. B. (1952). *Lancet*, **2**, 61.
[9] Stollerman, G. H. and Rusoff, J. H. (1952). *J.A.M.A.*, **150**, 1571.
[10] Perry, C. B. and Gillespie, W. A. (1954). *B.M.J.*, **2**, 729.
[11] Chamovitz, R., Catanzaro, F. J., Stetson, C. A. and Rammelkamp, C. H. (1954). *New Eng. J. Med.*, **251**, 466.
[12] Breese, B. B. (1953). *J.A.M.A.*, **152**, 10.
[13] Massell, B. F., Sturgis, G. P. Knobloch, J. D., Streeper, R. B., Hall, T. N. and Norcross, P. (1951). *J.A.M.A.*, **146**, 1469.
[14] Catanzaro, F. J., Brock, L., Chamovitz, R., Perry, W. D., Siegel, A. C., Stetson, C. A. Rammelkamp, C. H., Houser, H. B., Stolzer, B. L. and Wannamaker, W. L. (1955). *Ann. Int. Med.*, **42**, 345.

